# Neuroprotective Effects of Physical Activity: Evidence from Human and Animal Studies

**DOI:** 10.3389/fneur.2017.00188

**Published:** 2017-05-22

**Authors:** Sergio Chieffi, Giovanni Messina, Ines Villano, Antonietta Messina, Anna Valenzano, Fiorenzo Moscatelli, Monica Salerno, Alessio Sullo, Roberto Avola, Vincenzo Monda, Giuseppe Cibelli, Marcellino Monda

**Affiliations:** ^1^Department of Experimental Medicine, Università degli Studi della Campania “Luigi Vanvitelli”, Naples, Italy; ^2^Department of Clinical and Experimental Medicine, University of Foggia, Foggia, Italy; ^3^Department of Biomedical and Biotechnological Sciences, University of Catania, Catania, Italy

**Keywords:** physical activity and wellbeing, hippocampus, neurogenesis, growth factors, orexins

## Abstract

In the present article, we provide a review of current knowledge regarding the role played by physical activity (PA) in preventing age-related cognitive decline and reducing risk of dementia. The cognitive benefits of PA are highlighted by epidemiological, neuroimaging and behavioral studies. Epidemiological studies identified PA as an influential lifestyle factor in predicting rates of cognitive decline. Individuals physically active from midlife show a reduced later risk of cognitive impairment. Neuroimaging studies documented attenuation of age-related brain atrophy, and also increase of gray matter and white matter of brain areas, including frontal and temporal lobes. These structural changes are often associated with improved cognitive performance. Importantly, the brain regions that benefit from PA are also those regions that are often reported to be severely affected in dementia. Animal model studies provided significant information about biomechanisms that support exercise-enhanced neuroplasticity, such as angiogenesis and upregulation of growth factors. Among the growth factors, the brain-derived neurotrophic factor seems to play a significant role. Another putative factor that might contribute to beneficial effects of exercise is the neuropeptide orexin-A. The beneficial effects of PA may represent an important resource to hinder the cognitive decline associated with aging.

## Introduction

Clinical and epidemiological studies suggest that the physical activity (PA) can play an important and positive role in the prevention and treatment of age-related cognitive decline, as well as of a range of medical conditions, including type II diabetes, hypertension, heart disease, stroke, osteoporosis, cancers, and obesity. In support of the beneficial effects of PA on cognitive performance, there are a number of epidemiological, behavioral, and neuroimaging studies.

Physical activity was identified as an influential lifestyle factor in predicting rates of cognitive decline (1, 2) and the subsequent development of age-related neurodegenerative diseases such as Alzheimer’s disease (AD) (3, 4). Women who reported being physically active at any point over the life course, especially as teenagers, showed a lower likelihood of cognitive impairment in late life (1). Yaffe et al. (2) performed an interesting prospective study in which cognitive function of an older adult population was measured at baseline and subsequently. Over 8 years, 30% of the participants maintained cognitive function, 53% showed minor decline, and 16% had major cognitive decline. The authors (2) found that maintainers were more likely to engage in moderate to vigorous exercise compared to cognitive decliners. Neuroprotective effects of PA were also found by Larson et al. (5). They (5) followed a cohort of older adults (>65 years) over 6 years and found that regular exercise is associated with a delay in onset of dementia and AD. In particular, they reported a reduced incidence rate of dementia for persons who exercised three or more times a week compared with those who exercised fewer than three times per week. Subsequently, Buchman et al. (6) employed actigraphy to obtain an objective measure of total daily PA, circumventing in this way recall bias associated with traditional PA questionnaires. In the study (6) participated older adults [81.6 (7.12) years] who were followed for an average of 3.5 years. Participants in the lowest PA percentiles (10th percentile) had more than twofold higher risk of developing AD as compared to participants in the highest PA percentiles (90th percentile). Finally, two significant meta-analyses examined the association between PA and risk of dementia and found that PA was inversely associated with risk of dementia (3, 4).

Several neuroimaging studies also suggested a protective role of PA in preventing age-related decline related to brain atrophy. Comparing MRI images of older adults (60–79 years) collected before and after a 6-month aerobic fitness intervention, Colcombe et al. (7) observed significant increases in both gray matter and white matter (GM and WM) volumes as a function of fitness training. Interestingly, the increase of brain tissue volumes was primarily located in prefrontal and temporal cortices (7). In a subsequent research, Erickson and colleagues (8, 9) demonstrated that highly fit or aerobically trained participants showed preservation and increase of volume of the hippocampus, located in the inner (medial) region of the temporal lobe, and better performance on the spatial memory. Erickson et al. (9) reported an increase of the anterior hippocampus volume by 2% in older adults who followed 1-year aerobic exercise training, whereas there was a 1.4% decline in the control group that followed 1-year stretching intervention. This decline is comparable to the 1–2% shrinkage of hippocampus volume that was reported occurring annually in older adults (10). Other researches showed that increases in total PA were positively associated with increases in local GM volume in prefrontal and cingulate cortex (11) and greater WM integrity in the frontal and temporal lobes (12). Two interesting follow-up studies provided further support of a protective effect of PA against age-related decline (13, 14). In older adults (65 years old and older at baseline), greater amounts of PA were associated 9 years later with greater GM volume in prefrontal and temporal regions, including the hippocampus and entorhinal cortex (13). In turns, greater GM volume was related to a lower risk for experiencing cognitive impairment (13). In another study, participants were studied in midlife (early fifties) and re-examined on average 21 years later (14). Individuals who actively participated in PA at midlife tended to have larger total brain and GM volume, especially at level of the frontal lobes, in late life than sedentary persons (14). Rovio et al. (14) suggested that PA by activating the motor cortex localized in the frontal lobe also activated frontal structures related to cognitive functions, decreasing in this way the risk of dementia. Colcombe and Kramer (15) conducted an interesting meta-analytic study to examine the relationship between aerobic fitness training and cognition in healthy but sedentary older adults. They (15) found that fitness training had robust but selective benefits for cognition, with the largest benefits occurring for executive control processes. Some factors influenced the efficacy of the treatment: training duration (long-term training programs yield larger effect sizes); session duration within the training period (sessions exceeding 30 min had larger effect sizes); and the combination of strength and aerobic training regimens were more effective than aerobic exercise alone (15).

Then, the studies we have reported show that the frontal region is one of the brain regions that get more benefit from PA. Frontal areas subserve critical executive control processes, including the inhibition of irrelevant information (16–20). Flanker (21) and Stroop (22) tasks, and Digit Symbol Substitution Test (23) were employed to examine whether PA enhanced the ability in inhibiting irrelevant information in older adults. In the flanker task, participants were asked to respond to the direction of the central arrow while ignoring the two flanking arrows on either side (21); in the Stroop task, to respond manually to the color of ink in which the word was printed, rather than responding to the semantic meaning of the word (22); in the DDS, to match a number–symbol pair (probe) to a previously showed number–symbol pair (cue) (23). Highly fit or aerobically trained participants showed a better behavioral performance and greater task-related activity in prefrontal and parietal cortices, i.e., in regions consistently implicated in attentional selection and the resolution of response conflict.

## Mild Cognitive Impairment (MCI) and AD

The studies reported above support the view that PA seems not only to spare brain volume but also increase both GM and WM mainly in the prefrontal and temporal cortices. These brain areas play a critical role in cognitive functions. Prefrontal regions are associated with working memory and executive functions (24–28) and temporal lobes with long-term memory function (29–31). Interestingly, these regions are also those same regions that are often reported to deteriorate with aging (7, 23) and be severely affected in AD (32, 33).

Experimental evidence suggests a positive effect of aerobic exercise training on cognitive function in MCI and AD populations. MCI is a potential transitional stage between normal cognitive function and AD (34). MCI patients experience mainly memory loss to a greater extent than is expected for age and education, but do not meet criteria for AD (34). Two studies investigated the effects on cognitive performance of a 6-month aerobic exercise training in MCI participants (35, 36). Baker et al. (35) found that aerobic exercise had beneficial effects on cognitive performance of amnestic MCI participants (55–85 years). However, women improved on multiple tests of executive function, men only on a single test. Only women (70–80 years) with probable MCI participated to the study of Nagamatsu et al. (36). They showed an improvement of verbal memory and spatial memory. In another study, MCI (70–80 years) individuals participated to 1 year of a moderate-intensity aerobic walking program (37). The walking program was efficacious in improving memory and attention in women and memory in men, but only in those with better adherence (37).

Alzheimer’s disease is considered a neurodegenerative disease that brings about a variety of cognitive disorders and motor perturbations. In AD, subsequent to the loss of memory, the deficits carry over into the areas of language (aphasia), motion organization (apraxia), visual recognition (agnosia), and the executive functions (38). Also in the case of AD, several studies lend support for neuroprotective effects of PA. In the Kemoun et al.’s study (39), AD participants [81.8 (5.3) years] benefited from a 15-week PA program. There was an improvement in cognitive capacities and walking capacities (39). Conversely, the AD control group who did not practice any PA showed a deterioration of cognitive functions and walking capacities (39). In the study by Yágüez et al. (40), AD individuals [70.5 (8) years] who received 6-week exercise intervention showed significant improvements in sustained attention, visual memory, and a trend in working memory, whereas the AD control group deteriorated significantly in attention. Interestingly, PA seems to exert a beneficial effect on the hippocampus, a brain region particularly sensitive to age-related decay (9). Hippocampus shrinks with age (10) and its atrophy predicts shorter time-to-progression from MCI to AD (41). Erickson et al. (9) found an increase of anterior hippocampus size and better spatial memory performance with aerobic exercise intervention in older adults. Gains in hippocampal blood flow and memory performance were also observed by Chapman et al. (42) in healthy sedentary adults (57–75 years) with shorter term exercise (3 months).

## Animal Studies: Hippocampal Neurogenesis

Since the approach of human neuroscience is basically non-invasive, it does not allow direct measurement of exercise effects on the brain at the cellular and molecular level. To overcome this limitation, research employs animal models. In this context, the study of hippocampal changes produced by exercise attracted the interest of many research groups mainly for two reasons. First, as stated above, the hippocampus is a region sensitive to the beneficial effects of PA, but at the same time it is particularly vulnerable to age-related decay (7, 23). Note that the hippocampus is critically involved in memory processes (29, 30). Second, the hippocampus, along with the olfactory bulb, is the place in the adult in which new neurons are generated throughout life (43). Therefore, it is very important to accurately define the cellular and molecular mechanisms that support hippocampal neurogenesis. Some factors have been identified that seem to favor neurogenesis, including environmental enrichment, voluntary exercise, and associative learning (44–46). Early studies showed that the exposure to enriched environment increased neurogenesis in the dentate gyrus and improved also spatial memory performance of adult rodents (47, 48). However, in the enriched environment, more factors might contribute to enhance the neurogenesis, e.g., social, cognitive, and physical stimulations. van Praag et al. (44) tried to define the relative importance of some of these factors. They (44) assigned adult mice to various conditions, including enriched and standard housing, and voluntary and forced exercise. van Praag et al. (44) observed that voluntary exercise doubled the number of surviving newborn cells in amounts similar to enrichment condition. The authors (44) proposed that voluntary exercise was sufficient for enhanced neurogenesis in the adult mouse dentate gyrus. Hippocampal neurogenesis diminishes with aging (49), but this decrease may be partially opposed by exercise (46). Exercise-enhanced hippocampal neurogenesis and learning in aged mice (46). Interestingly, the morphology of new neurons did not differ between young and aged runners, suggesting that local hippocampal environment of the aged dentate gyrus is effective in sustaining neurogenesis (46).

A different line of research investigated the effects of hyppocampal lesion on behavioral performance. Clark et al. (50) irradiated with gamma rays the region of mice hippocampus reducing neurogenesis by 50%. The authors (50) observed that in non-irradiated animals running increased neurogenesis fourfold and gains in performance for the Morris water maze (spatial learning and memory), rotarod (motor performance), and contextual fear (conditioning). Conversely, irradiation, besides reducing neurogenesis, selectively eliminated gains in water maze performance that depends on hippocampus. The decrease in neurogenesis and cognitive skills, induced by irradiation, might be mitigated by exercise. Rats that received whole-brain irradiation and, following irradiation, were forced to perform exercise showed a significant amelioration of the impaired neurogenesis and cognition (51).

The morphological and functional changes in hippocampus produced by exercise likely depend on the contribution of different factors, including the enhancement of vascularization and upregulation of growth factors.

## Angiogenesis

Experimental evidence suggests that exercise increases angiogenesis (i.e., the growth of new blood vessels) in the hippocampus (46) and angiogenesis is closely linked to hippocampal neurogenesis (52). In a seminal study, Pereira et al. (52) used MRI imaging to measure cerebral blood changes related to exercise in mice and humans. Note that some studies reported the existence of a tight relationship in the brain between regional blood volume and angiogenesis (53). Pereira et al. (52) observed that in mice cerebral blood volume increased with exercise and presented a positive correlation with newly born cells (52). Also in humans (21–45 years) who participated to a 12-week exercise training, there was a significant increase of hippocampal dentate gyrus blood volume over baseline (52). Pereira et al. (52) proposed that the increase of hippocampal blood volume might be considered an *in vivo* correlate of neurogenesis.

## Growth Factors

Exercise upregulates expression of growth factors including brain-derived neurotrophic factor (BDNF), vascular endothelial growth factor (VEGF), and insulin-like growth factor-1 (IGF-1) (54, 55). Among these, the BDNF is considered to be the most important factor. A lot of studies suggest that the upregulation of BDNF plays a significant role in hippocampal neurogenesis, dendritic complexity, and synaptic plasticity (44–46, 56). Importantly, these structural changes in the hippocampus appeared associated with improved spatial learning and memory (44–46, 50, 57). Note that BDNF levels in serum and plasma are highly correlated with BDNF levels in the central nervous system, as BDNF freely crosses the blood–brain barrier (58). In humans, more researches reported that exercise increased BDNF concentrations serum suggesting a key role for this neurotrophic factor in enhancing hippocampal volume and cognitive function (9, 59, 60). Interestingly, circulating BDNF levels were reduced in patients with AD (61, 62). Furthermore, AD patients whose condition was rapidly declining have significantly lower serum BDNF concentrations than those whose condition was slowly declining (61, 63).

Angiogenesis factors, especially VEGF, are now known to have roles in neurogenesis and neuroprotection (64). Fabel et al. (65) showed that peripheral vascular endothelial VEGF is necessary for the effects of running on adult hippocampal neurogenesis. Peripheral blockade of VEGF abolished running-induced neurogenesis but had no detectable effect on baseline neurogenesis in non-running animals (65).

Exercise also increases the levels of IGF-1 in several brain structures, including the rat hippocampus (66). In aged rodents, circulating IGF-1 levels decrease (67). An increase of IGF-1 with exercise has been also reported in humans (68). Note that IGF-1 can cross (69) and increased levels of circulating IGF-1 result in increased IGF-1 levels in the brain (66). Blocking the entrance of circulating IGF-1 into the brain followed a complete inhibition of exercise-induced neurogesis in the hippocampus (70). A meta-analysis revealed a highly significant positive association between IGF-I levels and cognitive functioning in older adults (71). Patients with AD had significantly lower circulating IGF-1 levels than controls, and these levels were inversely correlated with cognitive impairment (72).

## Orexin-A and Orexin-B

Another factor that acting on the hippocampus might contribute to the beneficial effects of physical exercise on cognition is the orexin-A. The orexin-A/hypocretin-1 (OxA/Hcrt-1) and orexin-B/hypocretin-2 (OxB/Hcrt-2) are neuropeptides synthesized by a cluster of neurons in the lateral hypothalamus (73, 74). Orexins selectively act on two G protein-coupled receptors: the orexin 1 receptor (Ox1R), which has higher affinity to OxA, and the orexin 2 receptor (Ox2R), which has equal affinity to both OxA and OxB (73, 74). Ox1R and Ox2R are generally excitatory and mediate both acute and long-lasting effects (74). Orexinergic neurons receive a variety of signals related to environmental, physiological, and emotional stimuli and project broadly to the entire CNS (75). Orexinergic system is involved in regulating wakefulness and arousal, motivation and emotions, and motor and autonomic functions (76–84). Furthermore, orexinergic system may induce structural changes in the hippocampus influencing hippocampal learning and memory processes. Local dentate gyrus perfusion with OxA enhanced long-term potentiation (LTP) in anesthetized rats, suggesting that orexins positively regulated hippocampal synaptic plasticity (85). Conversely, the pretreatment with SB-334867, a specific Ox1R antagonist, blocked LTP (85) and impaired spatial memory in Morris water maze (86). In rats treated with Pentylenetetrazol that induces hippocampal atrophy and spatial learning and memory deficits, the administration of OxA enhanced hippocampal neurogenesis and attenuated learning and memory deficits (87).

Physical exercise produces an increase of OxA level in cerebrospinal fluid of rats (88), dogs (89), and cats (90). An increase of plasmatic OxA with exercise was reported in humans (91–95). The source of peripheral orexins is still unclear. Tsunematsu and Yamanaka (96) proposed that OxA might be directly released from the pituitary into the blood stream, or leaked from the cerebrospinal fluid, or produced by peripheral tissues, e.g., gastrointestinal tract and pancreas. Interestingly, OxA may rapidly cross the blood–brain barrier highly lipophilic (97). Taken together, the experimental data we have reported allow to hypothesize that the increase of OxA levels with exercise might contribute to improve cognition, enhancing hippocampal plasticity and function.

## Conclusion

In this review are discussed researches that support the view that PA is an effective tool for attenuating cognitive decline related to aging. PA would induce both morphological and functional changes of those regions that play central roles in successful everyday functioning, such as frontal and temporal cortices. In particular, exercise-induced hippocampal changes have attracted the interest of many researchers since the hippocampus, along with the olfactory bulb, is the place in the adult in which mammalian brain continues to generate new neurons throughout life. A better microcirculation and increased levels of growth factors seem to contribute to hippocampal neurogenesis. Another putative factor that might contribute to the beneficial effects of PA is the OxA. In favor of this hypothesis, there are the following observations: (1) hypothalamic orexinergic neurons have connections to hippocampus; (2) OxA enhances hippocampal neurogenesis and functions; and (3) OxA levels increase with exercise.

The regions that benefit from PA are also those that seem more vulnerable to aging, loading to a decline in a broad array of cognitive processes. In this context, PA may constitute a promising support for a neuroprotective effect against cognitive decline in MCI and AD. This is very important if we consider the continuous and progressive increase in the number of adults surviving to advanced age, and consequently the significant increase of health problems. Dementia represents the major threat of aging decline resulting in a considerable worsening of life quality not only of the patients but also of their family members, and in a dramatic increase of healthcare service costs. In this context, the PA may represent a simple, but effective and low cost, therapeutic intervention to improve neurocognitive functions. PA is accessible to most older adults and is not plagued by intolerable side effects that often occur with pharmaceutical treatments.

## Author Contributions

IV, AM, and AV: conceived the study, participated in its design, and wrote the manuscript. FM, MS, AS, RA, and VM: contributed to the conception and design of the study. SC, GC, MM, and GM: drafted the article and revised it critically for important intellectual content. GM: gave final approval of the version to be published. All authors read and approved the final manuscript.

## Conflict of Interest Statement

The authors declare that the research was conducted in the absence of any commercial or financial relationships that could be construed as a potential conflict of interest.
